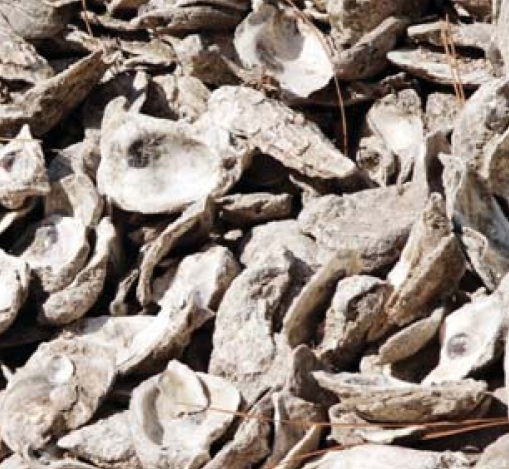# The Beat

**Published:** 2008-12

**Authors:** Erin E. Dooley

## Entré for Toxic Entrées?

When you eat red meat or cow’s milk, you also consume *N*-glycolylneuraminic acid (Neu5Gc), a sugar molecule found in those foods, researchers reported 5 years ago. New work by the same group, published online 29 October 2008 ahead of print in *Nature*, shows moreover that subtilase cytotoxin secreted by Shiga toxigenic *E. coli*—also found in red meat and cow’s milk—preferentially targets human cells carrying the Neu5Gc molecule. Humans were once presumed resistant to subtilase cytotoxin because they do not produce Neu5Gc. The new findings suggest, however, that eating contaminated meat can both expose people to foodborne illness and make them more vulnerable to it.

**Figure:**
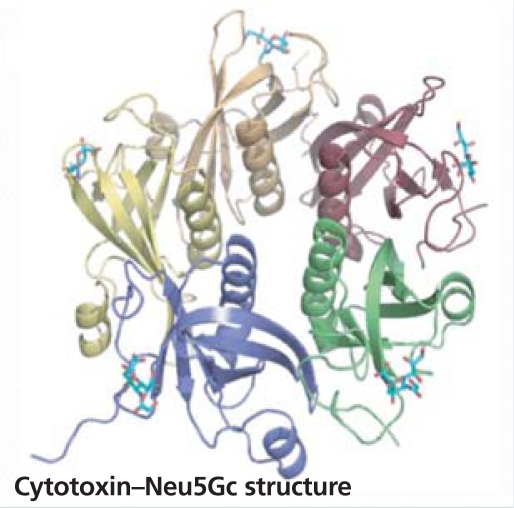
Cytotoxin–Neu5Gc structure

## Little Fish Add Up

A study in the 2008 edition of the *Annual Review of Environment and Resources* finds that “forage fish” such as anchovies and sardines make up 37% of all fish harvested each year and that 90% of this highly nutritious catch is used in animal feed. Forage fish are an inexpensive alternative to the plant-based foods sometimes used in animal feed. The study authors state that the use of these fish for animal feed competes with human consumption of them in some developing countries, where they provide a crucial source of protein and other beneficial nutrients. Overfishing of forage fish also harms populations of sea life that feed on them, such as cod, flounder, salmon, and tuna.

## New Measures for Worker Health

In November 2008, the American College of Occupational and Environmental Medicine and the Integrated Benefits Institute sponsored a national summit to promote health and productivity management (HPM), a practice in which employers measure the health of their employees and their productivity levels in order to determine health-related costs and the effects poor health can have on productivity. These measurements can guide the design of prevention and health programs specific to the needs of a given industry. A consensus statement from the meeting will be forthcoming.

## Island Nation’s Insurance Policy

The Intergovernment Panel on Climate Changes estimated in 2007 the global average sea level could rise as much as 2 feet by 2100. President-elect Mohamed Nasheed of the Maldives has taken that warning to heart. On 10 November 2008 he announced plans to create a “sovereign wealth fund” from tourism revenue to purchase land for relocation in the event the Maldivian homeland succumbs to rising sea levels. In an April 2008 book on the threat posed to his country by global warming, outgoing president Maumoon Abdul Gayoom wrote that the alternative to relocation—building protective walls around the 193 inhabited islands—was prohibitively expensive.

**Figure:**
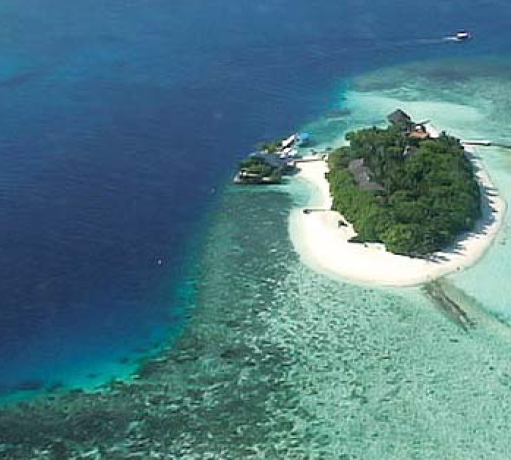
One of the islands of the Maldives

## Pregnancy Hypertension Risks Higher for Rural Women

There are several well-known risk factors for preeclampsia, in which pregnant women experience high blood pressure and proteinuria. At the November 2008 annual meeting of the American Society of Nephrology, researchers announced one more. In a study of 362,000 women, they found that living in a rural county carried a 56% increased risk. This increase in risk, which the researchers believe may be linked to maternal poverty or social isolation, was independent of other risk factors including poor prenatal care.

## Open Invitation to Oysters

Oysters purify water as they filter it through their bodies to obtain nutrition, with a single adult oyster filtering up to 60 gallons per day. Before the growth of the commercial shellfish industry in the last century, oyster beds along the Atlantic coast sometimes covered several square miles. Now oyster populations have been ravaged by overharvesting, pollution, and dredging. Environmental advocacy groups and government entities have teamed up to create an artificial reef in Wellfleet Harbor, Cape Cod, in an effort to improve water quality, strengthen the local marine ecosystem, and form a natural breakwater to protect the shoreline. The reef is composed of two 100-foot lengths of “cultch,” or crushed shells, banked by mesh bags loaded with shells. In a second phase of the project, concrete structures will be added to serve as habitat for juvenile oysters. As of November 2008, hundreds of oysters had begun populating the reef.

**Figure:**